# Supplementary material for: Warmer temperature accelerates senescence by modifying the aging-dependent changes in the mosquito transcriptome, altering immunity, metabolism, and DNA repair
Source: Immun Ageing. 2025 Dec 13;23:1. doi: 10.1186/s12979-025-00551-7 (PMC12781269; doi:10.1186/s12979-025-00551-7)
Supplement: Supplementary file 9 — Additional file 9. Table S2: Summary table of data depicted in Fig 8. [file 12979_2025_551_MOESM9_ESM.pdf]

Table S2. Warmer temperature and aging interactively shape the expression of genes involved in immunity. Direction of arrow indicates upregulation, downregulation, or no change.

	Gene (AGAP ID)	Interaction K-means Cluster Naïve	Interaction K-means Cluster HKEC	NAÏVE Warmer Temp	NAÏVE Aging	NAÏVE Temperature-Age Interaction	IMMUNE-INDUCED Warmer Temp	IMMUNE-INDUCED Aging	IMMUNE-INDUCED Temperature-Age Interaction
PATHOGEN RECOGNITION	***TEP1*** (AGAP010815)	n-4	i-9	↓	–	32°C reduces expression beyond 1 day of age.	↓	↓	32°C reduces expression beyond 1 day of age.
PATHOGEN RECOGNITION	***TEP4*** (AGAP010812)	n-5	i-9	↓	↑	32°C reduces expression beyond 1 day of age.	↓	↓	Aging-dependent decrease occurs faster at warmer temperatures.
PATHWAY SIGNALING AND ANTIMICROBIAL EFFECTORS	***UPD3A*** (AGAP013506)	n-2	i-1	↑	↑	Aging-dependent increase occurs faster at warmer temperatures.	↑	↑	Aging-dependent increase occurs faster at warmer temperatures.
PATHWAY SIGNALING AND ANTIMICROBIAL EFFECTORS	***JNK3*** (AGAP009460)	n-1	i-2	↑	↑	Aging-dependent increase occurs faster at warmer temperatures.	↑	↑	Warmer temperature does not increase expression at 1 day of age.
PATHWAY SIGNALING AND ANTIMICROBIAL EFFECTORS	***NOS*** (AGAP029502)	n-2	i-1	–	↑	At the oldest age, the warmest temperature increases expression.	↑	↑	After 1 day of age, aging-dependent decrease occurs faster at warmer temperatures.
PATHWAY SIGNALING AND ANTIMICROBIAL EFFECTORS	***DEF1*** (AGAP011294)	n-4	i-9	↓	↑	No interaction.	↓	↓	Aging-dependent decrease occurs faster at warmer temperatures.
MELANIZATION	***PPO6*** (AGAP004977)	n-10	i-9	↓	↓	Aging-dependent decrease occurs faster at warmer temperatures.	↓	↓	Aging-dependent decrease occurs faster at warmer temperatures.
MELANIZATION	***CLIPA5*** (AGAP011787)	n-8	i-6	–	↓	For some age groups, warmer temperature reduced expression.	–	↓	For some age groups, warmer temperature reduced expression.
