# Supplementary material for: Warmer temperature accelerates senescence by modifying the aging-dependent changes in the mosquito transcriptome, altering immunity, metabolism, and DNA repair
Source: Immun Ageing. 2025 Dec 13;23:1. doi: 10.1186/s12979-025-00551-7 (PMC12781269; doi:10.1186/s12979-025-00551-7)
Supplement: Supplementary file 10 — Additional file 10. Table S3: Summary table of data depicted in Fig 9. [file 12979_2025_551_MOESM10_ESM.pdf]

Table S3. Warmer temperature and aging interactively shape the expression of genes involved in metabolism and DNA repair. Direction of arrow indicates upregulation, downregulation, or no change.

	Gene (AGAP ID)	Interaction K-means Cluster Naïve	Interaction K-means Cluster HKEC	NAÏVE Warmer Temp	NAÏVE Aging	NAÏVE Temperature-Age Interaction	IMMUNE-INDUCED Warmer Temp	IMMUNE-INDUCED Aging	IMMUNE-INDUCED Temperature-Age Interaction
METABOLISM	**NADH dehydrogenase (ubiquinone) Fe-S protein 8** (AGAP001711)	n-7	i-7	↑	↓	Warmer temperature does not increase expression at 1 day of age.	↑	↓	Warmer temperature does not increase expression at 1 day of age.
METABOLISM	**6-phosphofructokinase (*PFK*)** (AGAP007642)	n-7	i-7	↑	↓	The warming-based increase in expression is amplified at 15 days of age.	↑	↓	The warming-based increase in expression is amplified at 15 days of age.
METABOLISM	**Pyruvate dehydrogenase phosphatase regulatory subunit** (AGAP002217)	n-7	i-7	↑	↓	Warmer temperature decreased expression at 1 day of age.	↑	↓	Warmer temperature decreased expression at 1 day of age.
METABOLISM	**Isocitrate dehydrogenase (NAD+)** (AGAP002192)	n-7	i-7	↑	↓	Warmer temperature decreased expression at 1 day of age.	↑	↓	Warmer temperature decreased expression at 1 day of age.
DNA REPAIR	**DNA helicase MCM8** (AGAP002580)	n-6	i-3	↓	↑	The warming-based increase only occurs beyond 1 day of age.	↓	↑	The warming-based increase only occurs beyond 1 day of age.
DNA REPAIR	**DNA ligase 1** (AGAP009222)	n-5	i-3	↓	↑	The warming-based increase only occurs beyond 1 day of age.	↓	↑	The warming-based increase only occurs beyond 1 day of age.
DNA REPAIR	**DNA repair protein Rad62** (AGAP010060)	n-6	i-4	↓	↑	The warming-based increase only occurs beyond 1 day of age.	↓	↑	The warming-based increase only occurs beyond 1 day of age.
DNA REPAIR	**DNA mismatch repair protein MSH4** (AGAP012245)	n-5	i-3	↓	↑	The warming-based increase only occurs beyond 1 day of age.	↓	↑	The warming-based increase only occurs beyond 1 day of age.
