# Supplementary figures and images for: Warmer temperature accelerates senescence by modifying the aging-dependent changes in the mosquito transcriptome, altering immunity, metabolism, and DNA repair
Source: Immun Ageing. 2025 Dec 13;23:1. doi: 10.1186/s12979-025-00551-7 (PMC12781269; doi:10.1186/s12979-025-00551-7)

Figure 1

A

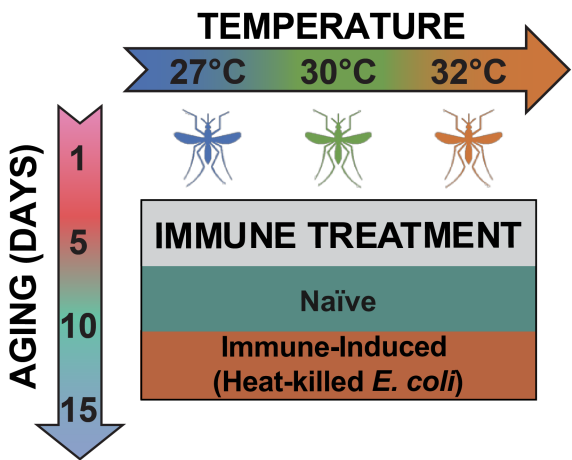

B

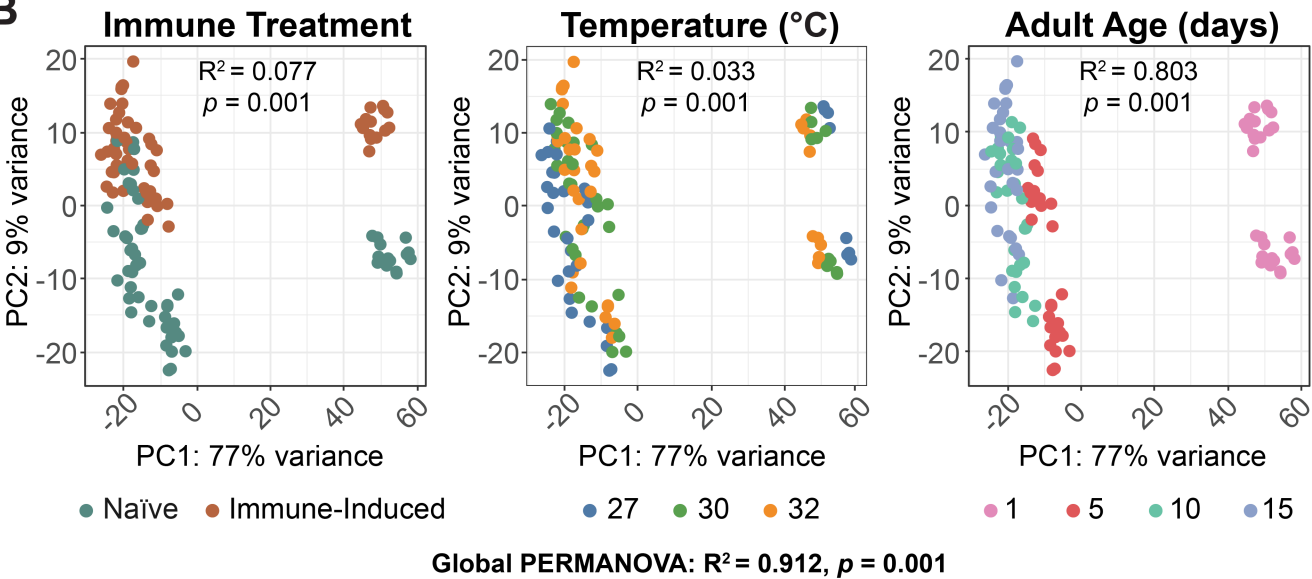

Figure 2

A

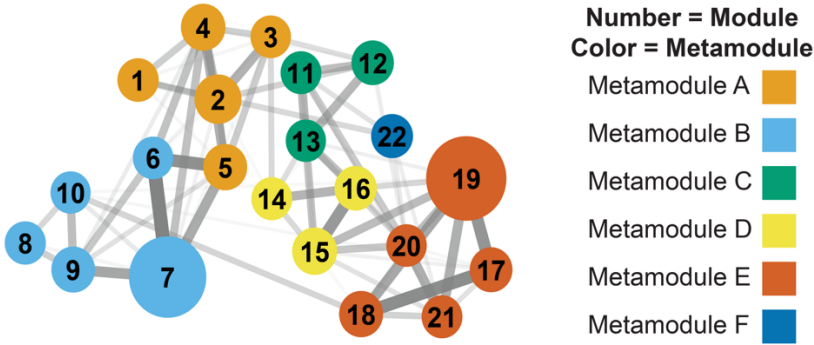

B

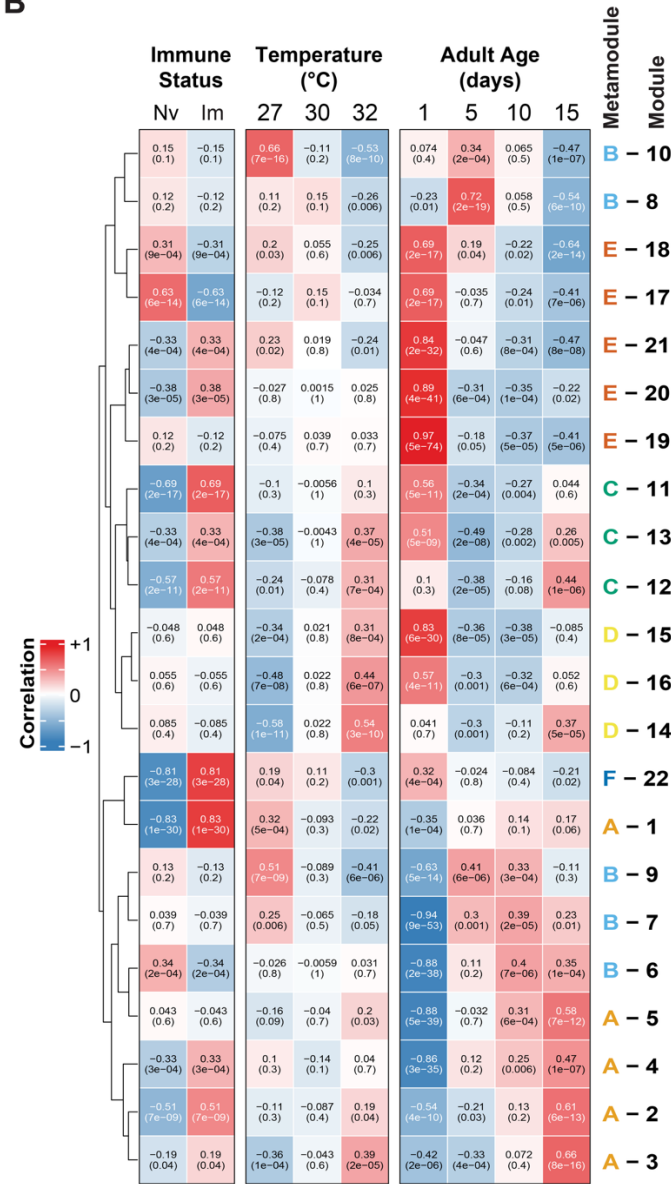

C

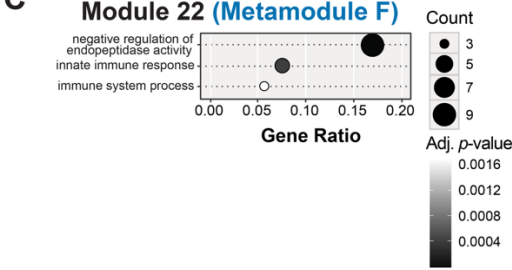

D

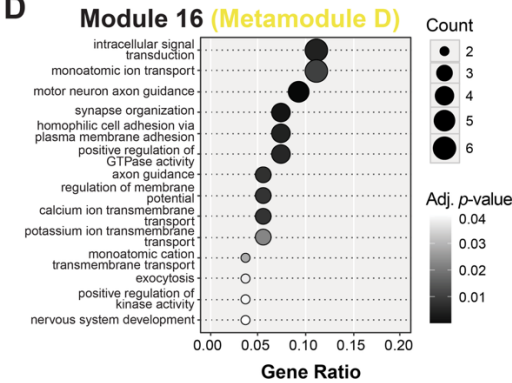

E

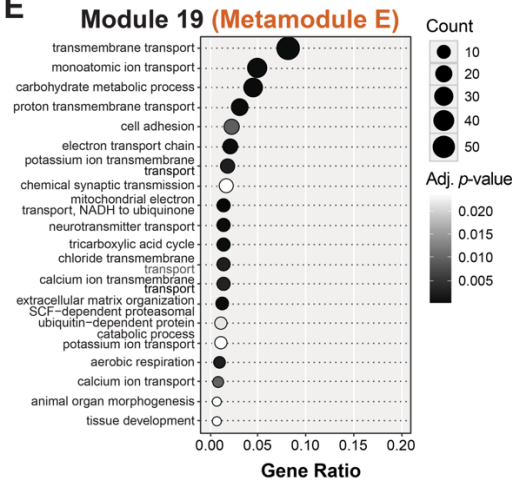

Figure 3

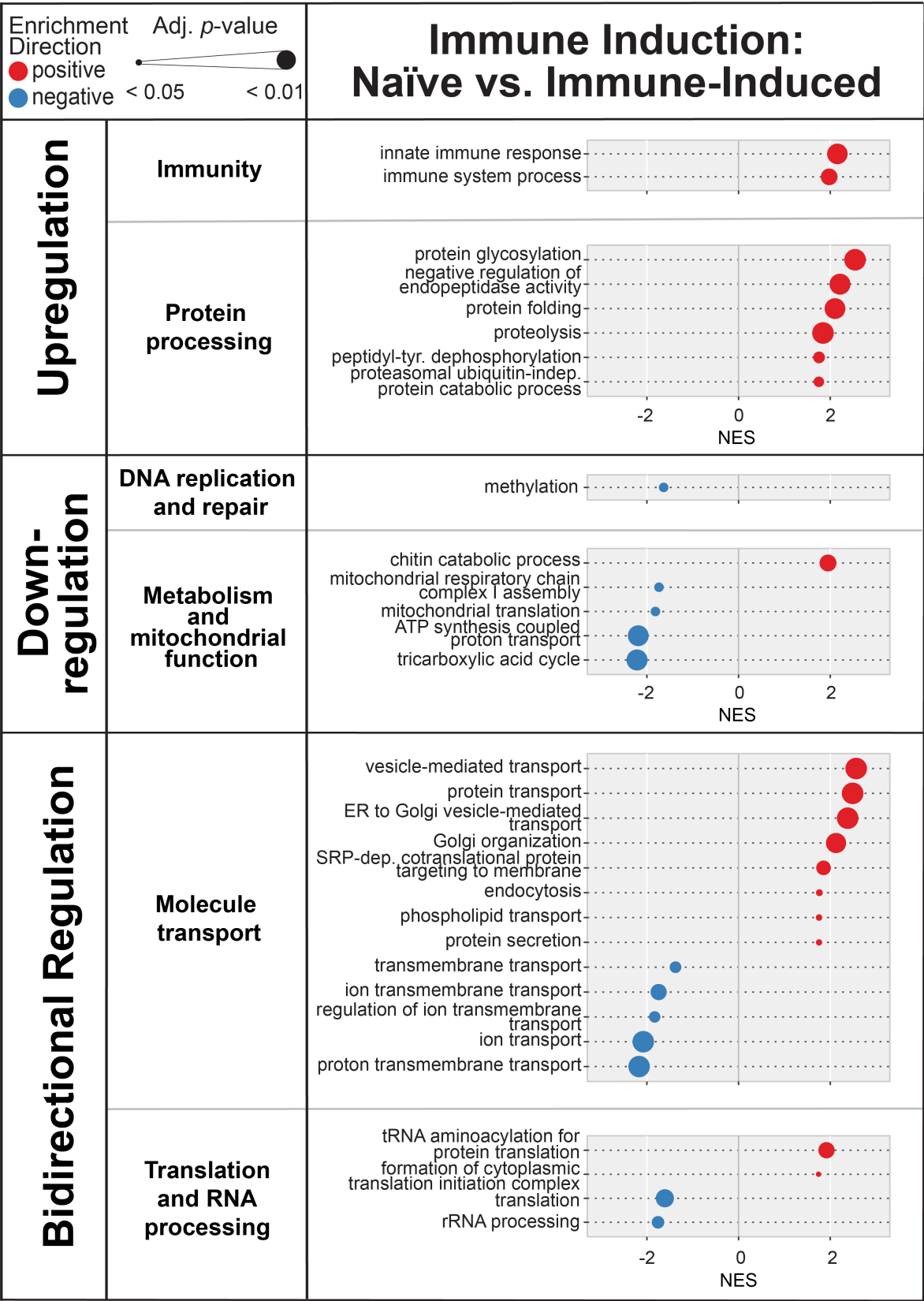

Figure 4

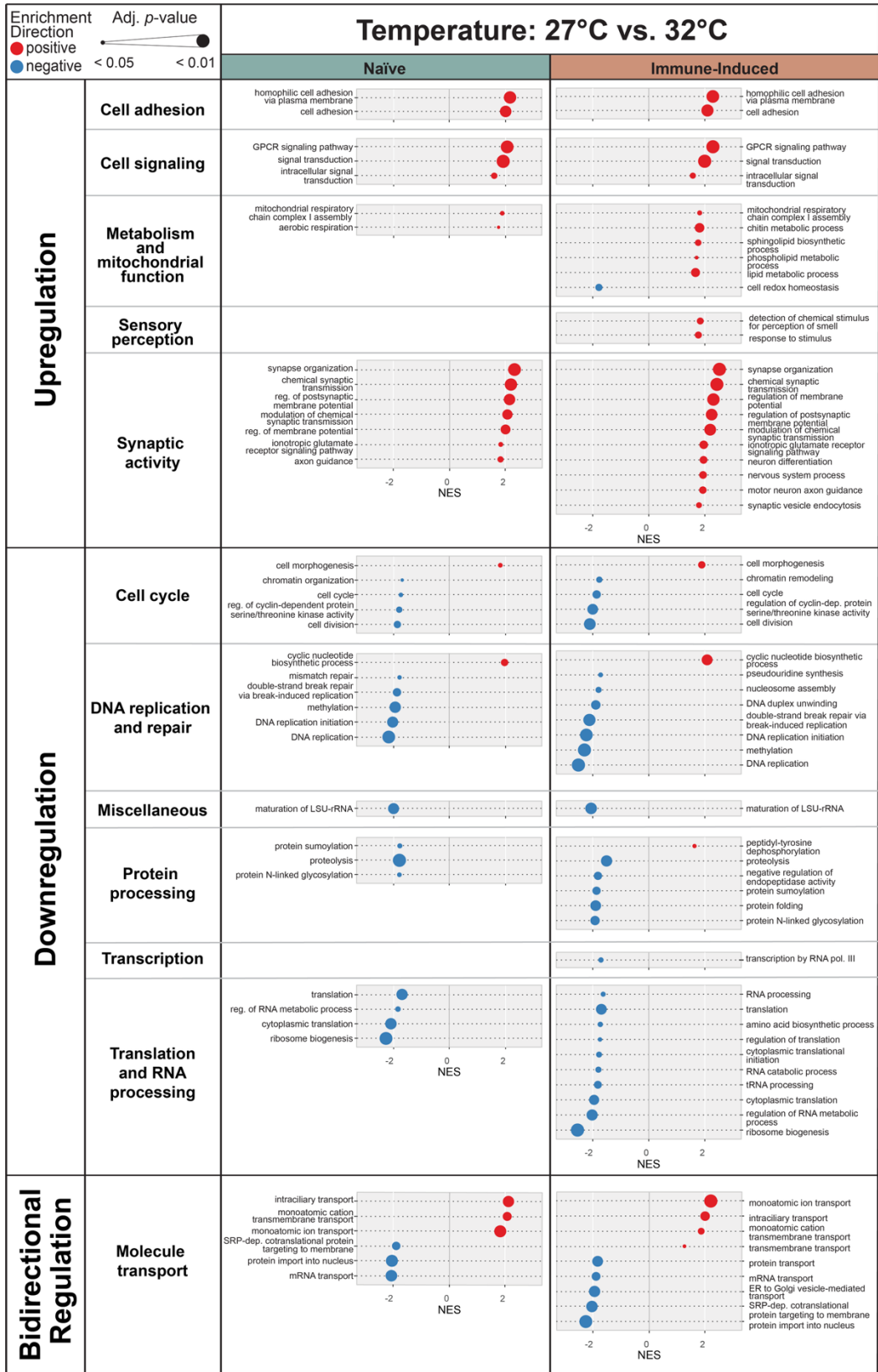

Figure 5

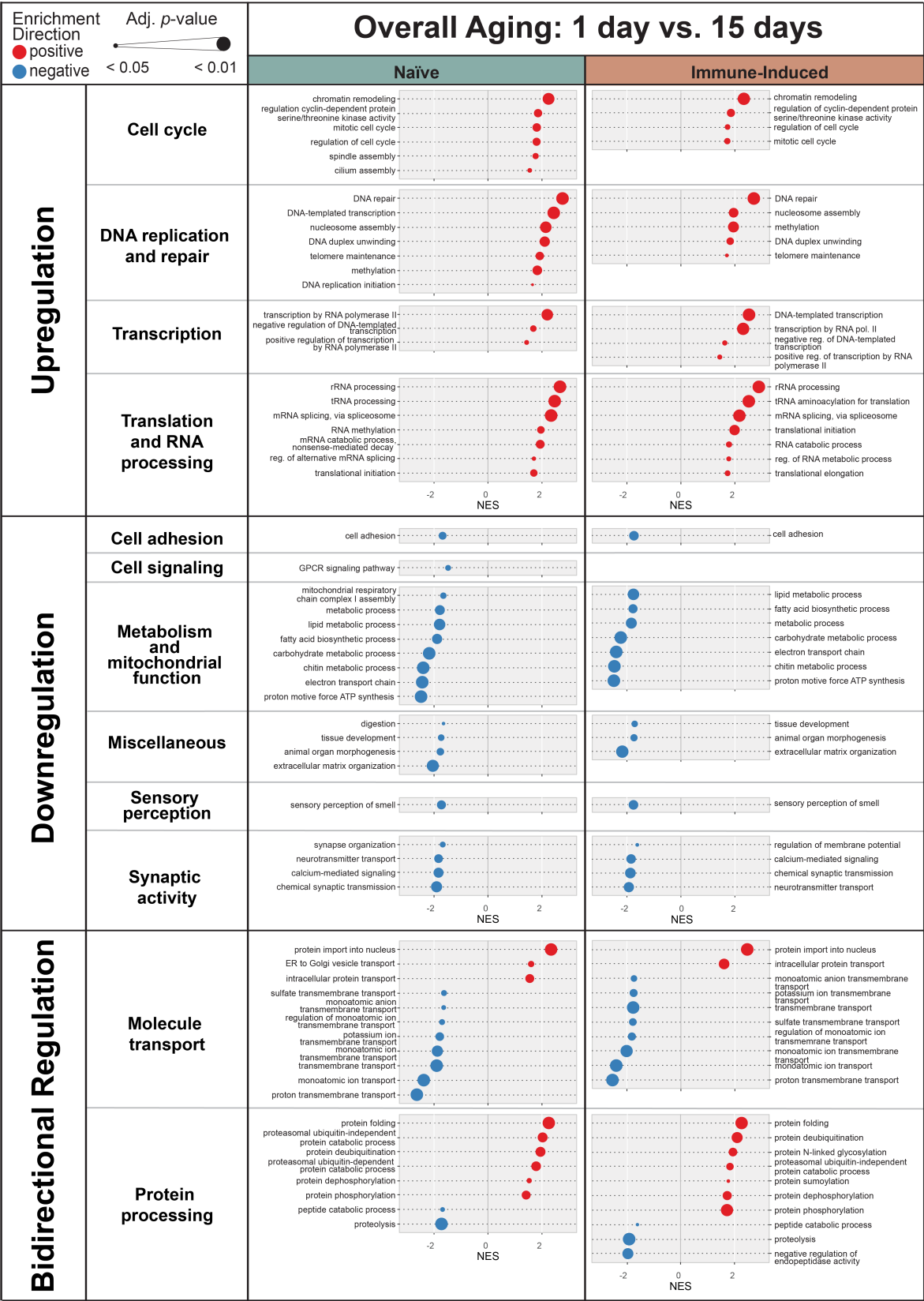

Figure 6

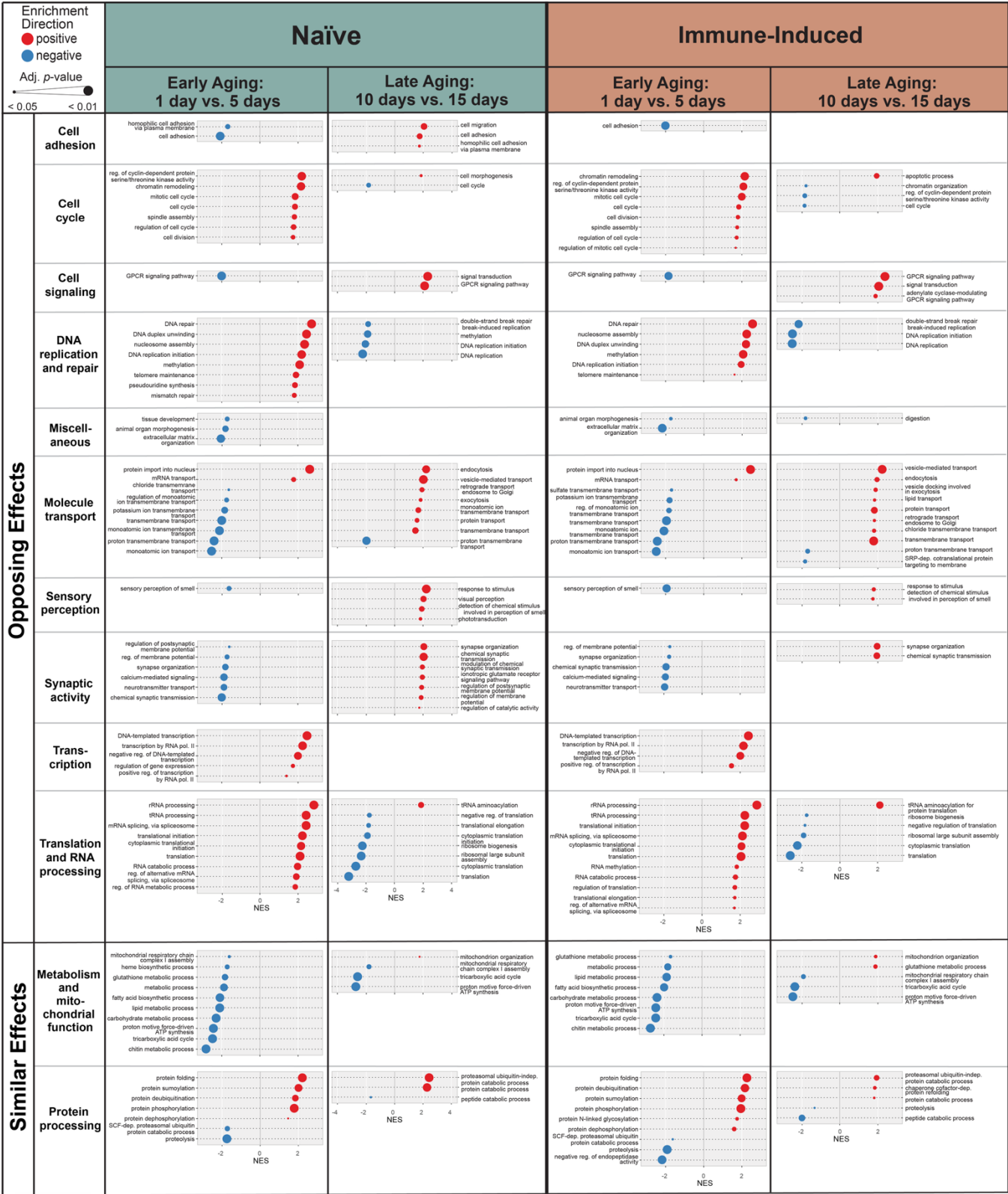

Figure 7

A

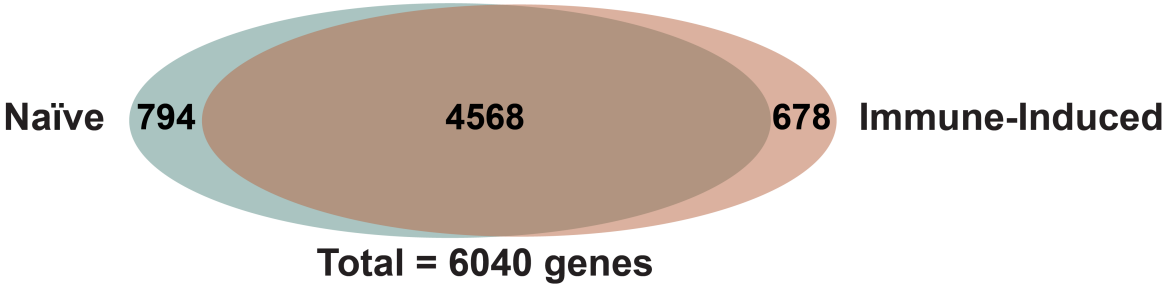

B

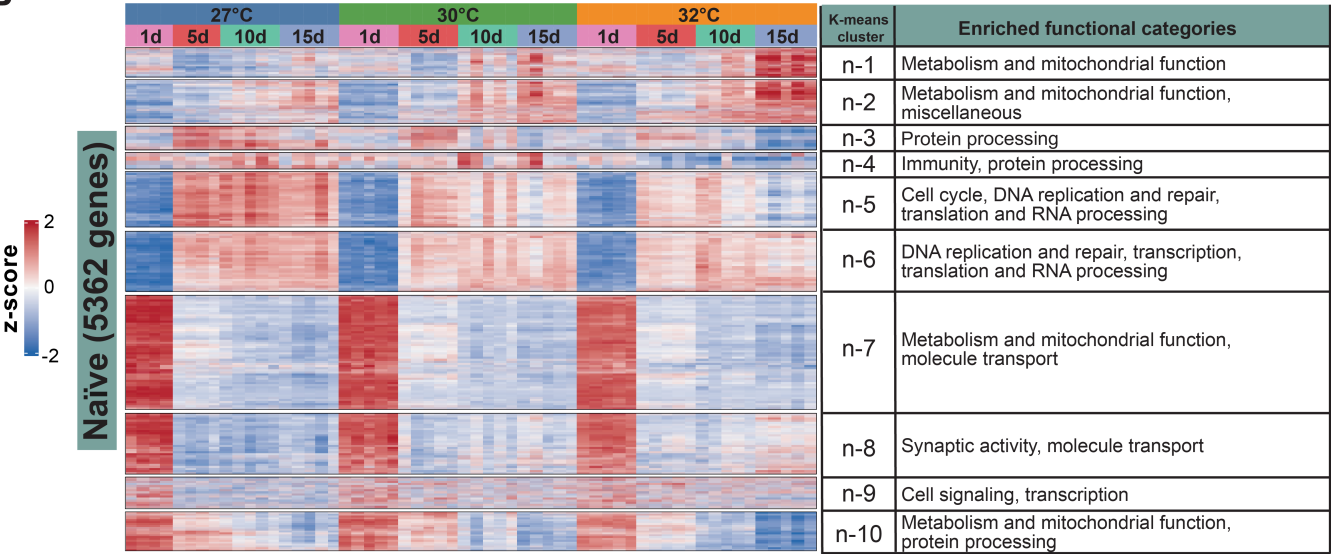

C

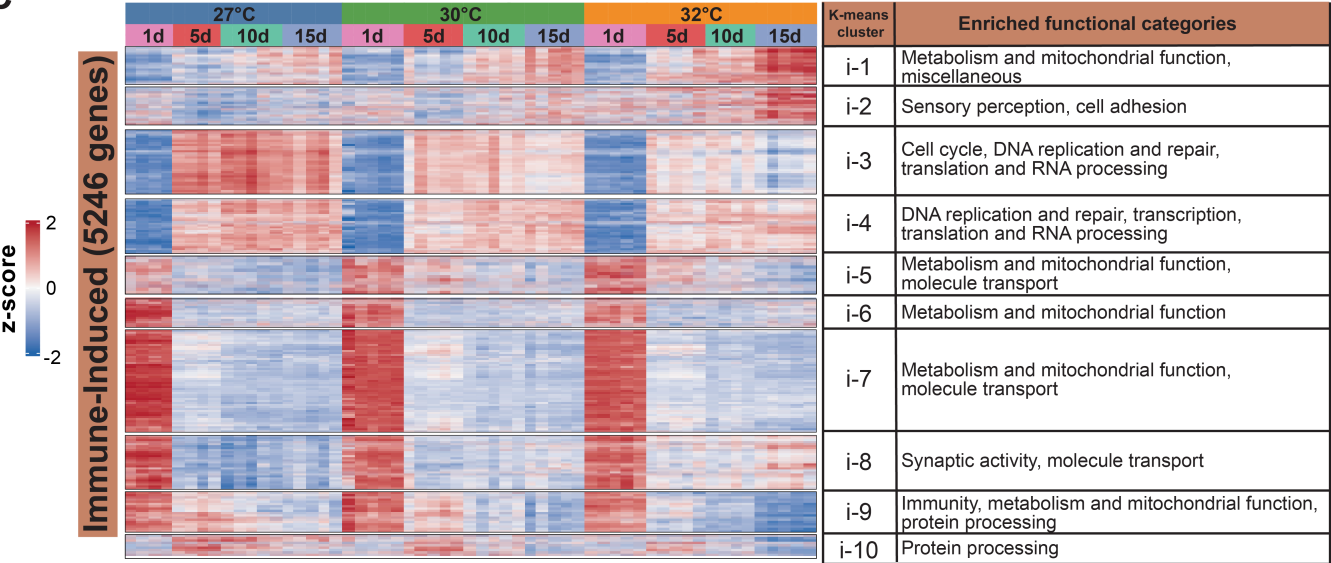

Figure 8

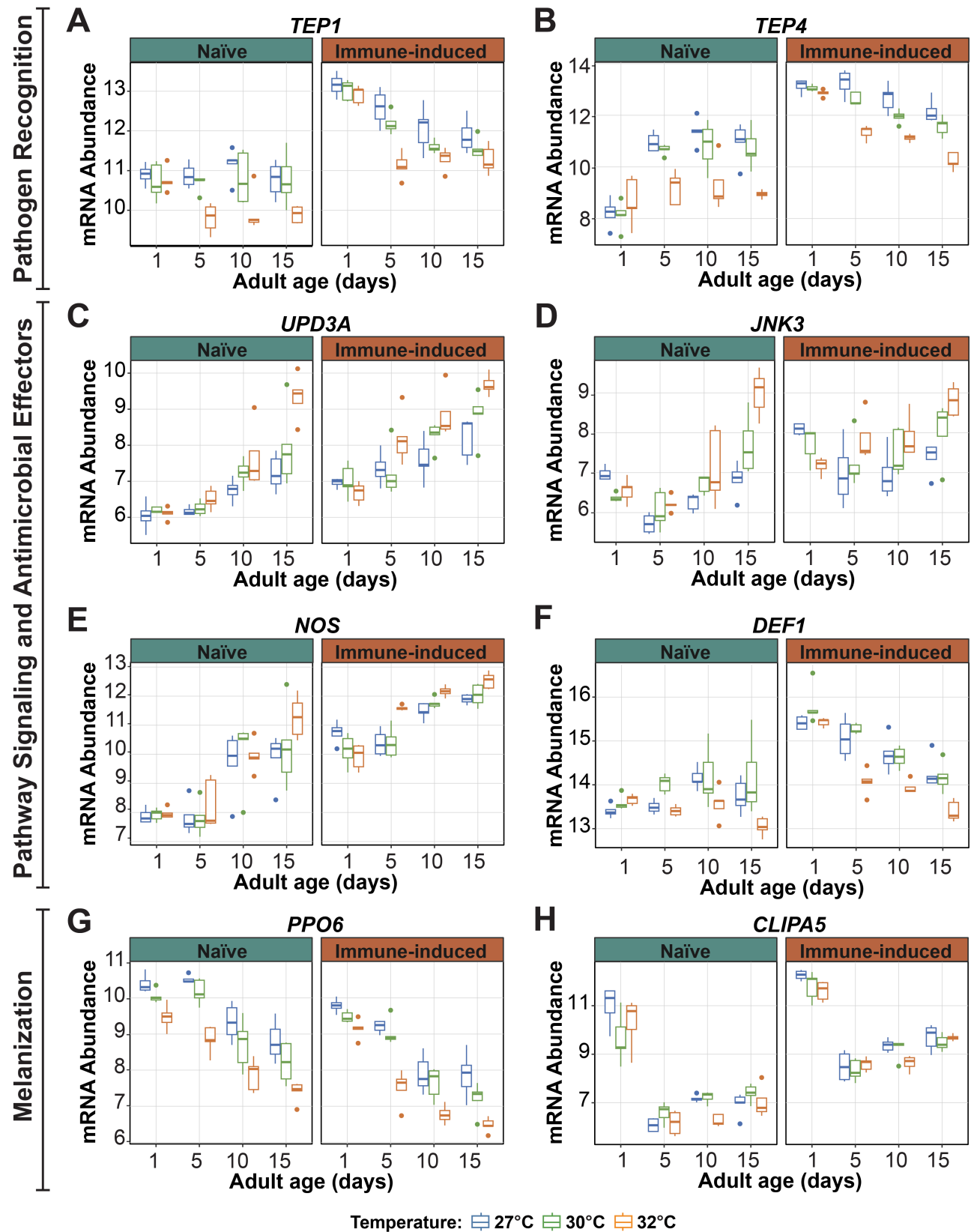

Figure 9

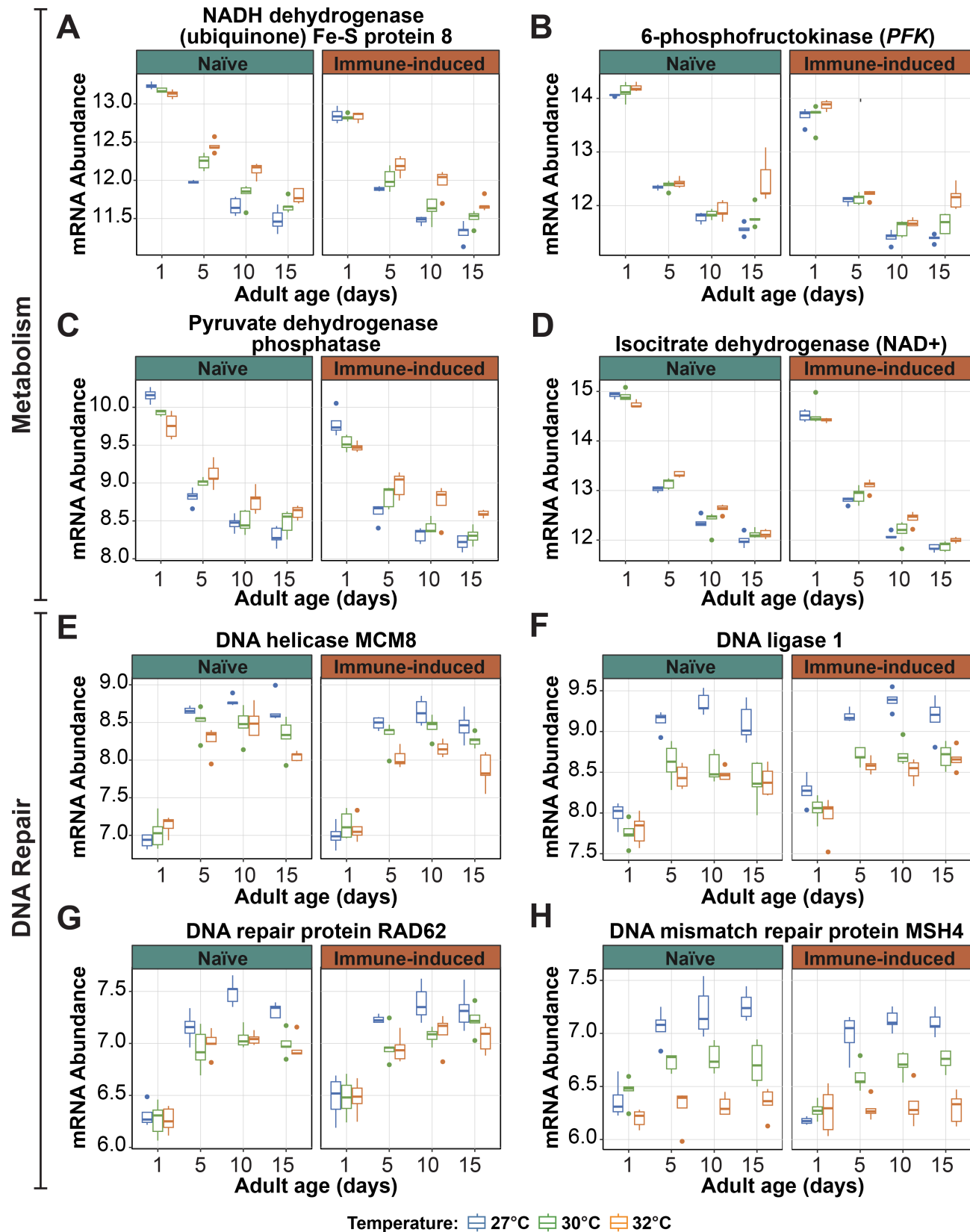

Figure 10

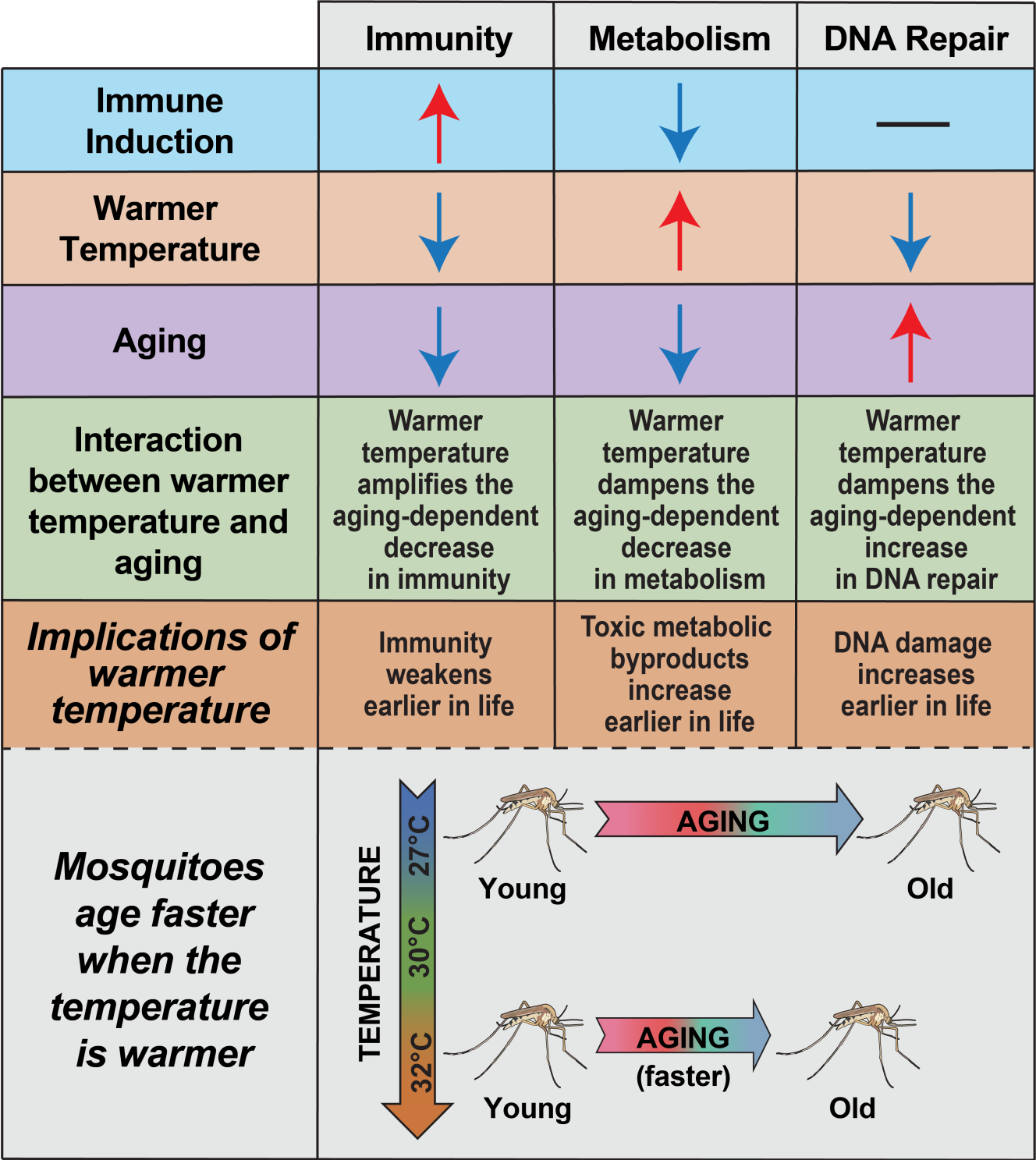

Supplement: Supplementary file 11 — Additional file 11. File S7: Higher resolution versions of the figures. [file 12979_2025_551_MOESM11_ESM.pdf]
